# Mood trajectories showing resilience and recovery in young people during and after the COVID-19 pandemic

**DOI:** 10.1038/s41598-026-39808-6

**Published:** 2026-02-14

**Authors:** Yara J. Toenders, Kayla H. Green, Lysanne W. te Brinke, Sophie W. Sweijen, Suzanne van de Groep, Matthijs Fakkel, Danielle Remmerswaal, Eveline A. Crone

**Affiliations:** 1 Erasmus School of Social and Behavioral Sciences, Erasmus University Rotterdam, Burgemeester Oudlaan 50, 3062PA Rotterdam, The Netherlands; 2 Department of Cognitive Sciences and Psychology, Amrita Vishwa Vidyapeetham, Amritapuri, India; 3 Developmental and Educational Psychology, Leiden University, Leiden, The Netherlands

**Keywords:** Mood, Adolescence, COVID-19 pandemic, Trajectories, Human behaviour, Risk factors

## Abstract

The COVID-19 pandemic greatly affected daily life of young people but not all youth were affected in the same way. We studied mood trajectories in 363 young people (10–29 years) in the Rotterdam area in the Netherlands across eight longitudinal waves spanning four years to test for resilience and recovery profiles during and after the COVID-19 pandemic. A four-class model using growth mixture modelling confirmed that some adolescents were highly affected with slow recovery (16%), some were moderately affected (27%), and there were two patterns of stable low to moderate mood level groups (24% and 33% respectively). Those that were highly affected in negative mood were characterized by more academic stress, more executive functioning problems, and lower sense of belonging. The same factors were predictive and protective for recovery trends of vigor. These results confirm that group averages do not fully capture the variability within adolescence and unravel protective factors that may guide future interventions in case of a new crisis.

**Supplementary Information:**

The online version contains supplementary material available at 10.1038/s41598-026-39808-6.

## Introduction

The coronavirus disease 2019 (COVID-19) not only massively affected physical health worldwide, it also impacted mental health and wellbeing^1^. In March 2020, the WHO officially declared the COVID-19 infection spread a pandemic. As a result of pandemic-related policy measures such as social distancing, school closures, and lockdowns, people’s social environments became more restricted. The imposed restrictions and following social isolation resulted in feelings of loneliness, a decline in well-being, and an increase in psychopathological symptoms such as anxiety and depression^2–6^. Adolescents and young adults might have been particularly hit by these social restrictions, since adolescence is a sensitive period for social-affective development and many mental health difficulties develop during adolescence^5,7,8^.

Initial research on mental health during the pandemic showed that children and adolescents reported an increase in internalizing symptoms, both in the general population as in a cohort with existing mental health difficulties^6,9^. Moreover, parents reported an increase in internalizing symptoms for children in the general population. This increase in anxiety and depression was associated with the extent to which adolescents perceived their lifestyle to be affected by the COVID-19 pandemic^10^. Thus, it is important to not only examine the general response to a stressful situation, but also the individual differences.

Many individual trajectories of mood and resilience, the ability to maintain or regain well-being when experiencing adversity, might exist^11–13^. Some adolescents might be affected by a stressful situation initially, but recover swiftly, whereas others suffer from the situation more long-term. Others might not be affected by the adverse experience, or even benefit from it, as suggested by studies demonstrating lower mental health problems or no change in wellbeing^14–16^. Indeed, also after the school closures and lockdown, youth reported increased depression and anxiety compared to pre-COVID-19^17^. However, not all adolescents had similar mental health problems after the to the pandemic-related restrictions were introduced, suggesting that some adolescents are more resilient than others (Fernández et al.,^18^; Pedrini et al.,^19^). Factors that might have acted as a potential buffer for mental health difficulties included higher social or family support, physical activity, and the use of positive coping strategies^20^.

An important question concerns whether there are dissociable trajectories of how adolescents are impacted by pandemic restrictions, and what defined recovery and resilience^11^. Earlier studies into the effect of the prolonged stress of the COVID-19 pandemic suggest that these differential trajectories of mental health, mood and resilience exist^21^. In adults from the UK, it was shown that symptoms of depression and anxiety overall decreased after the first lockdown^22^, suggesting signs of resilience. However, risk factors such as being female, being younger, and having lower income were associated with higher depression and anxiety even when there was an overall decrease observed. Person-centered approaches in young people showed different resilience profiles during the pandemic, with protective factors such as male gender and being younger in a sample of 10–19 years old^23–25^. Higher negative mood, how one feels at a specific time, has been associated with lower mental health^26^. Therefore, prior studies not only examined mental health trajectories, but also mood trajectories. In adolescents, there were some initial increases in vigor found, potentially due to less demands that they might have experienced^27^. The trajectories of potential recovery after the COVID-19 pandemic in adolescents and the risk and resilience factors associated with these trajectories are not yet known. It is important to unravel these trajectories, that earlier person-centered studies hinted towards, as these individual differences can be precursors for mental health problems.

Therefore, in this longitudinal preregistered study (https://osf.io/y35gd) we aimed to examine different trajectories of resiliency, through measuring adolescent mood for a prolonged period of 4 years, during and after the COVID-19 pandemic (May 2020 to December 2023). We investigated how individual trajectories of mood vary in subgroups of adolescents and whether there are identifiable factors that distinguish resilient adolescents, those who were able to maintain their wellbeing or bounce back to being well, from their peers. This research question was investigated in the longitudinal Urban Rotterdam project. In this study adolescents aged 10–29 years old^28^filled out online questionnaires every 6 months for 8 waves. The first timepoint was collected two months after the start of COVID-19 in May 2020, and the last wave was post-pandemic in December 2023. It is hypothesized that four trajectories will be recognized^29,30^. The largest group is expected to show resiliency, displaying a decrease in negative mood post pandemic, with depression and tension at the highest levels during the first 2 timepoints (May 2020 and November 2020).

Next, we studied the effect of age on the development of mood throughout and after the pandemic, given that older adolescents are expected to display more negative mood^31^. Lastly, we examined whether the developmental trajectories of mood differ in protective or risk factors such as academic stress, academic drop-out, sense of belonging, and executive functioning^29^. The COVID-19 pandemic and the level of resilience might influence the school performance of young people (academic stress and academic drop-out), while sense of belonging and executive functioning might function as protective factors, increasing the likelihood that someone will be resilient^32–35^.

## Methods

### Participants and procedure

Participants from the Urban Rotterdam project were included in this study. Participants were Dutch young people aged 10 to 25 at their first timepoint and took part in the 8-wave longitudinal study (https://erasmus-synclab.nl/project/the-urban-rotterdam-project/). All participants signed an informed consent. Additionally, parents of adolescents aged 15 or below signed parental consent for participation in the study. From wave 4 onwards, new additional participants were recruited and included at each wave (Supplementary Figure S1). Participants were recruited through social media, schools, and our website. Participants filled out online questionnaires through the Qualtrics platform. In the first two waves, participants were asked to fill out daily questionnaires during a period of two weeks, and from wave 3 onwards participants only took part in one questionnaire per wave (approximately 30 min per wave). They received a monetary reward for participation, a total of €15 at wave 1 and 2, and €10 from wave 3 onwards. The Ethics Committee of the Erasmus School of Social and Behavioural Sciences at Erasmus University Rotterdam approved the study (application 20–036).

### COVID-19 pandemic

The first wave of data collection took place in May 2020 during the first lockdown of the COVID-19 pandemic, during which social isolation restrictions were put into place. The second, third and fourth wave took place in December 2020, May 2021 and November 2021 respectively, at which time there were also lockdown restrictions in the Netherlands. The fifth to eighth waves were collected from May 2022 to December 2023, which are considered post-COVID (after lockdown restrictions had ended).

### Measures

#### Mood

At each wave, participants filled out 19 items of a Dutch translation of 3 subscales from the Profiles of Mood States (POMS) questionnaire^36^. This is a self-report to assess current mood (in this study specifically tension, depression, and vigor). Participants were asked to what degree 19 adjectives described their current mood on a 5-point Likert scale, ranging from 0 (not at all) to 4 (extremely). In the first 2 waves, daily mood was collected for two consecutive weeks, however, the current study only used the mood data from the first day in line with the latter waves. A sum score was calculated for tension (ranged between 0 and 24), depression (ranged between 0 and 32) and vigor (ranged between 0 and 20). Items were summed to calculate the subscale scores. A sum score for negative mood, based on tension and depression was calculated (ranged between 0 and 56). Vigor was examined separately as an assessment of positive mood and resilience. The Cronbach alpha per subscale was 0.87, 0.92, 0.81, 0.94 for tension, depression, vigor and negative mood respectively. Mood during the first three waves has previously been reported^31,37^.

#### Executive functioning

Executive functioning was measured with the webexec questionnaire^38^. This questionnaire consists of 6 questions to measure problems with executive functioning such as ‘Do you find it difficult to keep your attention on a specific task?’, that are answered on a 4-point Likert scale. Items were summed to calculate the total score, with a higher score indicating more problems with executive functioning. The stability of this measure over time was examined and a mean score was calculated across all timepoints available by the participant. The intraclass coefficient (ICC) was 0.64 [0.60–0.68].

#### Academic stress

The exhaustion subscale of the Dutch version of the Maslach Burnout Inventory, called Utrecht Burnout Scale (UBOS) was used to measure academic stress at timepoint 2 to 8^39^. This subscale consists of 5 questions such as ‘*At the end of a school/study day I feel empt*y’ that are rated on a 7-point Likert scale. A higher score indicates higher levels of exhaustion. A mean score was calculated across all timepoints available by the participant. The ICC was 0.55 [0.50–0.60].

#### Sense of belonging

The sense of belonging at school or studies was assessed using the school factor from the Social Connectedness Scale at wave 6^40^. This subscale consists of 9 questions such as ‘*I feel at ease about how I should behave at my school/studies/work’* that are answered on a 4-point Likert scale. A higher score indicates a higher sense of belonging. A sum score of sense of belonging at wave 6 was calculated.

#### Academic drop-out

At each wave the participants filled out their current education. Based on this information a variable was derived that displayed whether participants dropped out of school, repeated a year, or continued their education. For the current study, participants were divided into 2 groups: continued as normal or other cases (which consisted of dropping out of school or repeating a year).

### Statistical analyses

First, the development of mood throughout and after the COVID-19 pandemic was studied in the whole sample using general additive mixed models (GAMMs). This was a deviation from the preregistration since development of mood was found to be non-linear. Mood was used as the outcome variable, with wave as the predictor. A random effect for subject was added. Bayesian Information Criteria (BIC) was used to assess model fit to select the best *k* (representing the maximum degrees of freedom of the smoothing splines). The ‘mgcv’ package in R was used for the GAMM analyses^41^.

Next, to test whether there is a general developmental effect on mood development during and after the COVID-19 pandemic, a linear mixed model was tested with time and (linear and quadratic) age. Gender was included as a covariate, and a random intercept was added per participant.

Since large individual differences have been observed in the development of mood, we examined different trajectories of mood development throughout the COVID-19 pandemic using growth mixture modelling (GMM)^42^. For the GMM a strategy with 3 steps was used to handle the missing data. In the first step, only participants with data on at least a half of the timepoints were included. This means that participants were included in at least 4 waves of the Urban Rotterdam study. The remaining missing data was imputed using predictive mean matching from the mice package in R^43^. We included age at first wave and gender and all POMS subscales across waves in the imputation model. Five imputed datasets with 50 iterations each were created, the first completed dataset was used in the subsequent analyses. The next step was then to do a sensitivity analysis for which only participants with (almost) complete data were included to test whether the imputation did not majorly alter the results. Only participants with POMS data on at least 7 timepoints were included for this sensitivity check. Lastly, a more lenient approach was taken, for which participants with data on at least 2 timepoints were available were included. The results of these sensitivity analyses can be found in the Supplemental Material.

The mood trajectories were separately identified based on the negative (tension and depression) and positive (vigor) subscales of the POMS. GMM can be used to find trends over time in longitudinal data, while accounting for between-person effects. The ‘lcmm’ package in R was utilized for the analyses^44^. We executed models with an increasing number of trajectories until non-convergence or the proportion of participants per trajectory was below 5%. The fit of the consecutive models were compared using the Akaike Information Criterion (AIC), the Bayesian Information Criterion (BIC), and entropy. We then tested what GMM had a better fit with the Lo-Mendell-Rubin test (LMR).

Next, the different groups (with distant mood trajectories throughout and after the COVID-19 pandemic) were compared on demographic and protective or risk factors. Group membership was predicted based on age, gender, education, psychiatric diagnosis at inclusion, and academic drop-out. Next, the trajectories were compared, by looking at academic stress (burnout), sense of belonging, and mean executive functioning across waves. Analyses for differences on baseline measures were Bonferroni corrected (4 analyses), as well as the analyses for differences on protective or risk factors (3 analyses).

## Results

Data from 363 participants who participated in at least 4 timepoints were included, leading to a total of 2904 observations of mood after imputation (Table 1).

**Table 1:** Overview of demographic and protective or risk factors across waves before imputation. Mean (SD) are being displayed unless otherwise specified.

*N*	Mean (SD)
	363
**Demographics**	
Age at first inclusion	17.9 (3.2)
Gender (M/F/non-binary/other) n (%)	76 (21%) / 279 (77%) / 3 (1%) / 5 (1%)
Ethnicity at first inclusion	
Dutch	243 (67%)
Multiple ethnicities including Dutch	66 (18%)
Education at first inclusion	
Primary school	2 (1%)
High school VMBO	6 (2%)
High school HAVO/VWO	185 (51%)
Vocational education (mbo)	45 (12%)
Higher education (hbo/university)	99 (27%)
No education	25 (7%)
Academic drop-out (continued, repeated year, drop-out) n (%)	277 (76%) / 12 (3%) / 38 (11%)
**Mood**	
Tension	7.2 (4.3)
Depression	6.6 (5.2)
Negative (sum tension and depression)	13.8 (9.1)
Vigor	10.0 (3.3)
**Protective or risk factors**	
Utrecht Burnout Scale	3.5 (1.3)
Executive functioning	13.5 (3.9)
Sense of belonging	27.4 (5.2)

### Development of mood during and after the COVID-19 pandemic

**Fig. 1:**
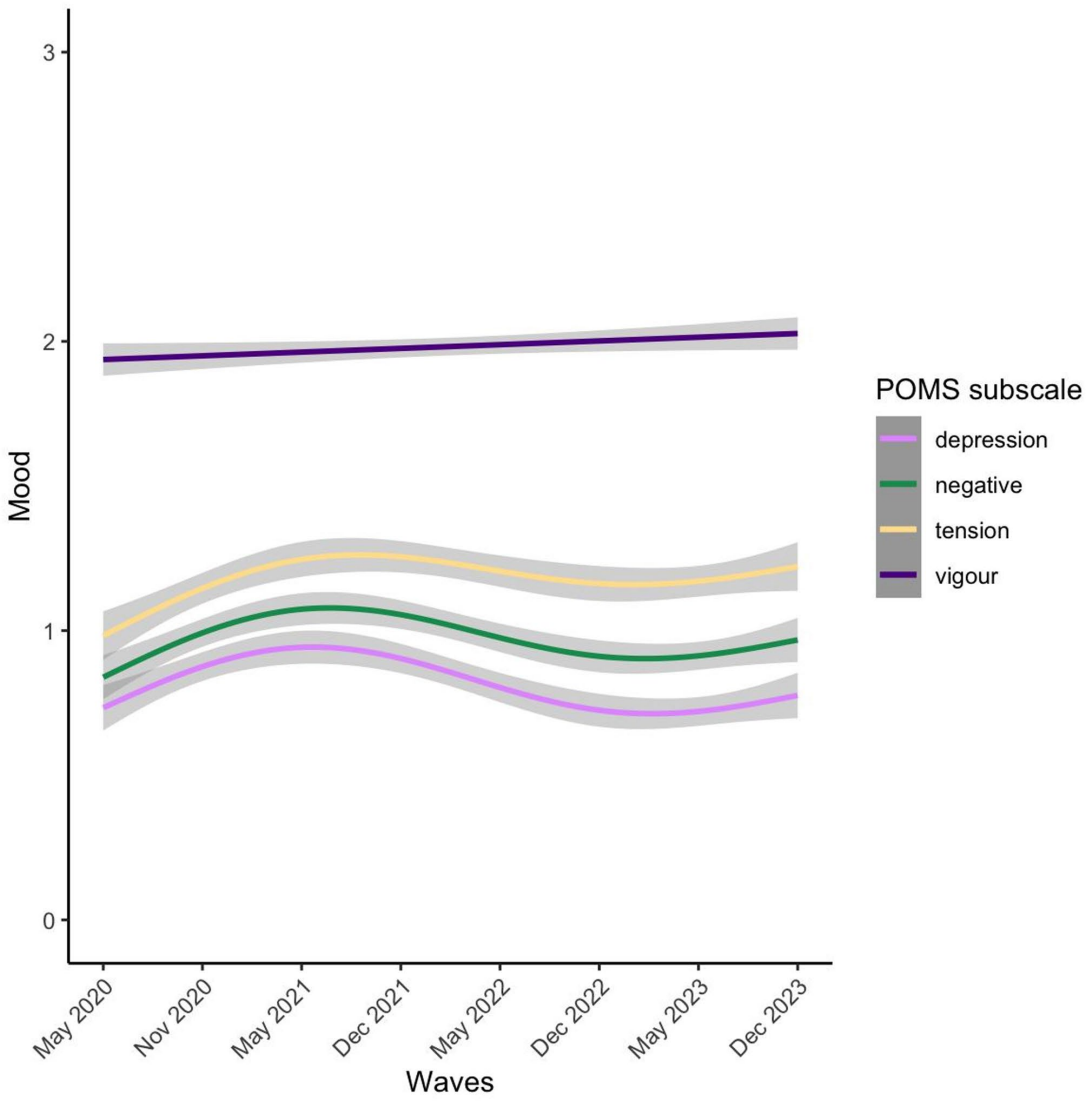
Development of mood (per subscale) during and after the COVID-19 pandemic. The y-axis presents the mood sum score per subscale (averaged for visualization purposes). Negative mood was the summed value of the depression and tension subscales. The x-axis represents the different waves, from May 2020 to December 2023.

Across the entire sample of adolescents, the development of mood during the pandemic was examined using general additive mixed models (GAMMs). It was found that both the negative subscales (tension and depression) and the positive subscale of the POMS (vigor) changed over time (*F* = 16.99, *p*<.001, *k* = 4; *F* = 19.86, *p*<.001, *k* = 4; *F* = 7.08, *p*=.02, *k* = 3 respectively; Fig. 1 and Supplemental Table S1). Tension and depression peaked around wave 3 and 4 (May and December 2021), followed by a subsequent decrease. This decrease was larger for depression than tension. For all subsequent analyses, the two negative subscales tension and depression were combined into one negative subscale because of their high correlation (*r*=.79, Supplementary Figure S2). Vigor showed a linear increase throughout the waves. Negative mood and vigor were only moderately negatively correlated (*r*=-.44), therefore they were treated as separate variables in the analyses.

### Effect of age on the development of mood during and after the COVID-19 pandemic

**Fig. 2:**
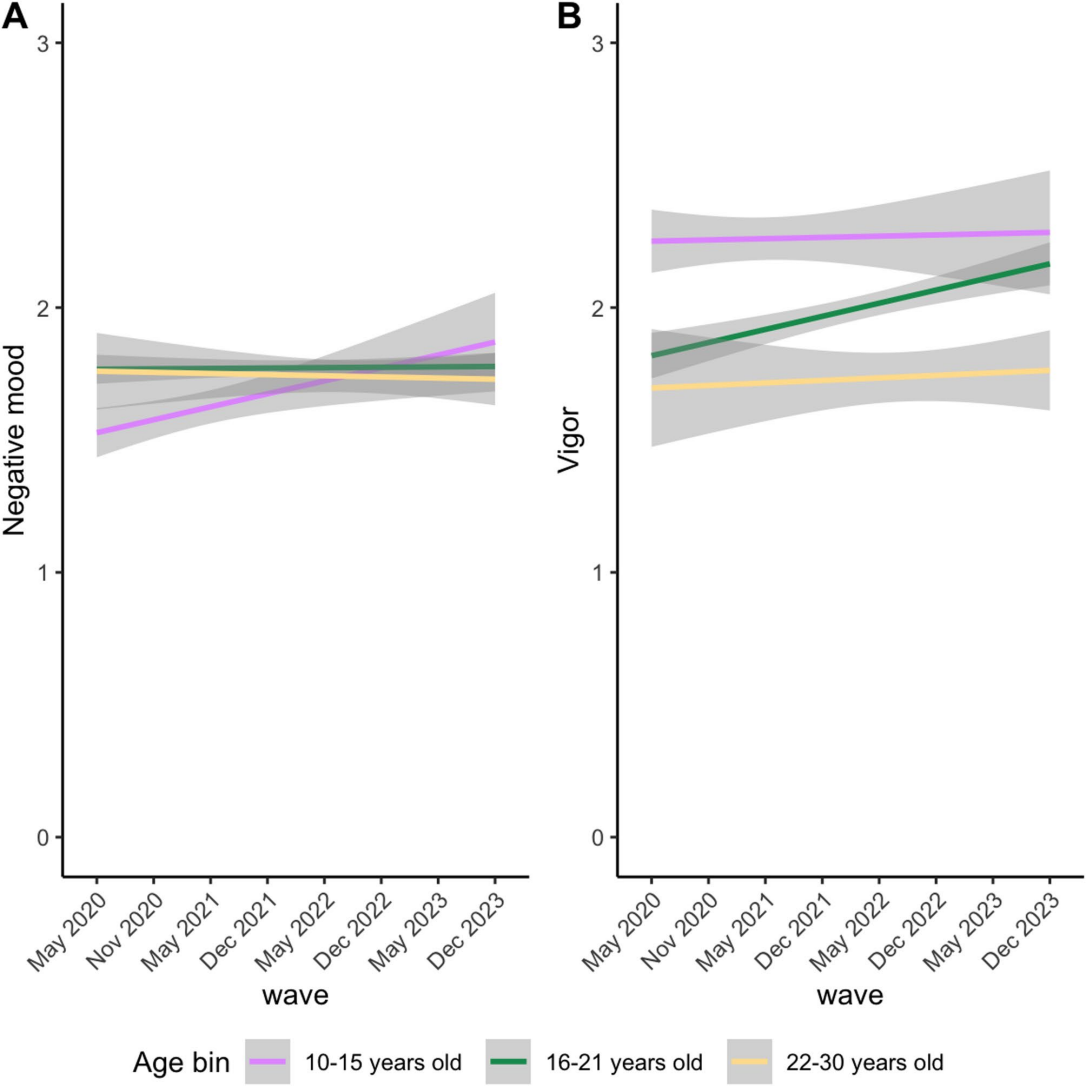
Interaction between age and wave on mood (A: negative mood, B: vigor) during and after the COVID-19 pandemic. The y-axis presents the mood sum score per subscale (averaged for visualization). Age was divided into bins for visualization purposes only. Wave and age showed an interaction effect for negative mood, with younger ages being less affected by the COVID-19 pandemic, but older ages showing a recovering effect post-COVID. A main effect for age was found for vigor, with younger ages showing more positive mood.

Before analysing the classes, we first tested the possible effects of age on the development of mood during and after the COVID-19 pandemic using linear mixed effect models. It was found that age and wave showed an interaction for negative mood (*F* = 6.47, *p* = < 0.001), but not vigor (*F* = 2.12, *p*=.08). Vigor only showed a main effect of age, with younger ages showing higher vigor (*F* = 29.72, *p* = < 0.001). Figure 2 presents the age effects across waves. For visualization purposes, age was divided into three bins (early adolescence, late adolescence and young adulthood). As can be seen in Fig. 2, adolescents with higher ages showed a more negative mood during COVID-19, but they recovered post-COVID, whereas younger adolescents were less affected but did not show a post-COVID decline in negative mood.

### Different trajectories of mood during and after COVID-19

**Fig. 3:**
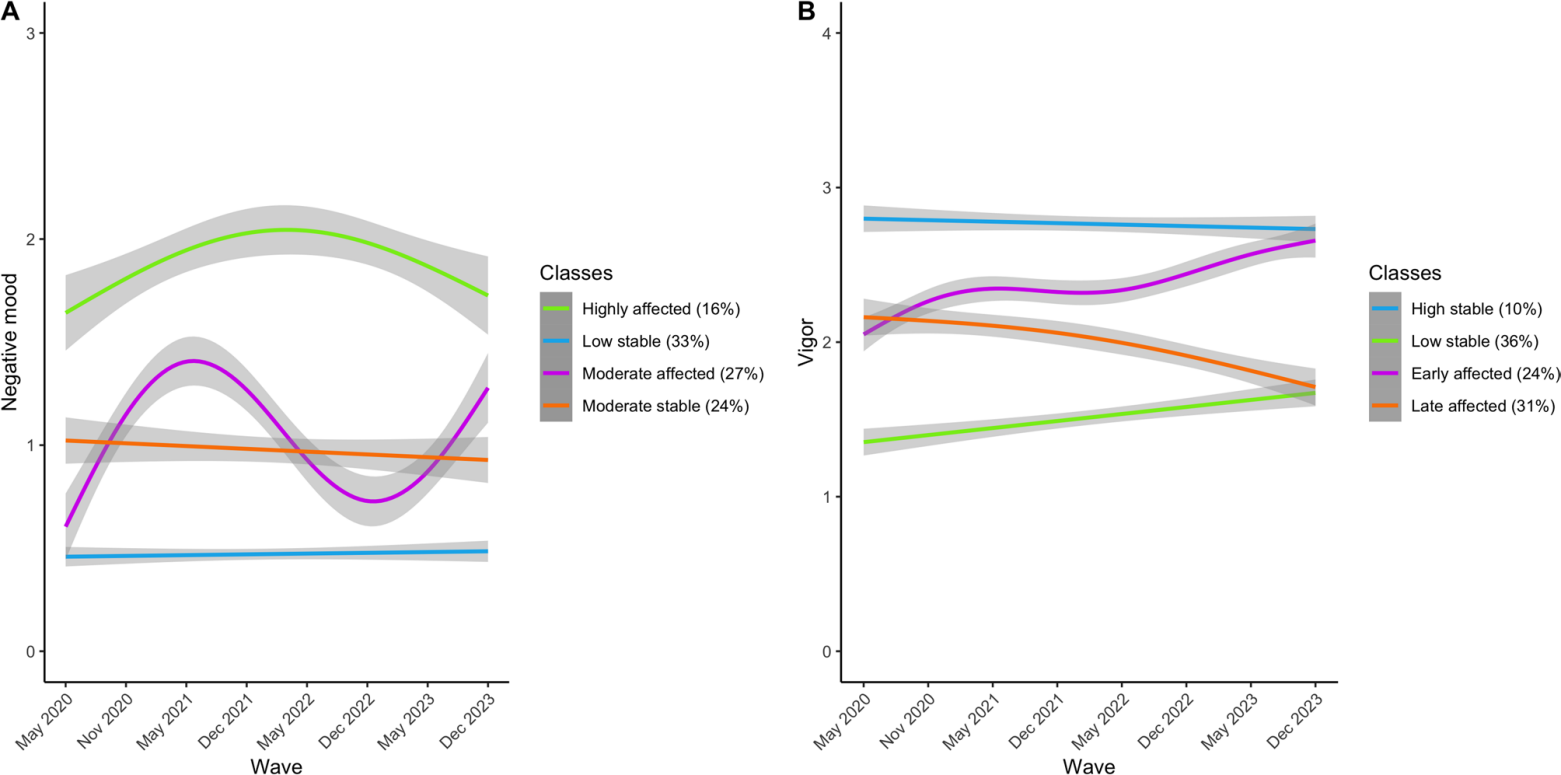
Four different trajectories of (left) negative mood and (right) vigor during and after the COVID-19 pandemic. The y-axis on the left presents the negative mood per wave per class (averaged for visualization purposes). The y-axis on the right presents vigor per wave per class (averaged for visualization purposes). The trajectories per class were fit using GAM with 3 knots, linear regression, GAM with 4 knots and linear regression (respectively per class for negative mood) and linear regression, linear regression, GAM with 5 knots and GAM with 3 knots (respectively per class for vigor).

Next, we addressed the question whether classes could be distinguished for negative mood trajectories across the waves. For negative mood, a model with 4 different trajectories was considered the best fit based on AIC, entropy, and Lo-Mendell-Rubin test (Supplementary Table S2, Fig. 3A). We identified two classes that did not show a change in mood during the COVID-19 pandemic. They differed on the average negative mood, resulting in a ‘low negative mood’ (Low stable – 33%) and a ‘moderate negative mood’ class (Moderate stable – 24%). The third class (Highly affected – 16%) was characterized by high negative mood, especially during the COVID-19 pandemic, peaking at the third and fourth wave (May 2021 and November 2021), with a small recovering effect afterwards. However, in this group negative mood remained at early pandemic level. The final class (Moderate affected – 27%) showed a similar increase in negative mood during the COVID-19 pandemic, and a subsequent decline, returning to similar levels as the moderate stable class. Similar results were found in the more stringent and lenient dataset (Supplementary Table S3, Supplementary Figure S3-S5).

Based on vigor, a model with four different classes was also identified as the optimal solution (Supplementary Table S2). As can be seen in Fig. 3B, the first class was characterized by high stable levels of vigor (High stable, *n* = 58 (10%)), the second and largest class by low vigor, that slightly improved after the COVID-19 pandemic (Low stable, *n* = 119 (36%)). The third class showed lower vigor in the earlier waves but improving levels of vigor comparable to the level of the high stable class in the later waves (Early affected, *n* = 99 (24%)) and lastly the fourth class was characterized by high levels of vigor in the earlier waves but decreasing levels of vigor in the later waves, to the level of the stable low class (Late affected, *n =* 87 (*31*%)). Similar results were found in the more stringent and lenient dataset (Supplemental Material).

### Identifiable factors of trajectories of mood during and after the COVID-19 pandemic

**Fig. 4:**
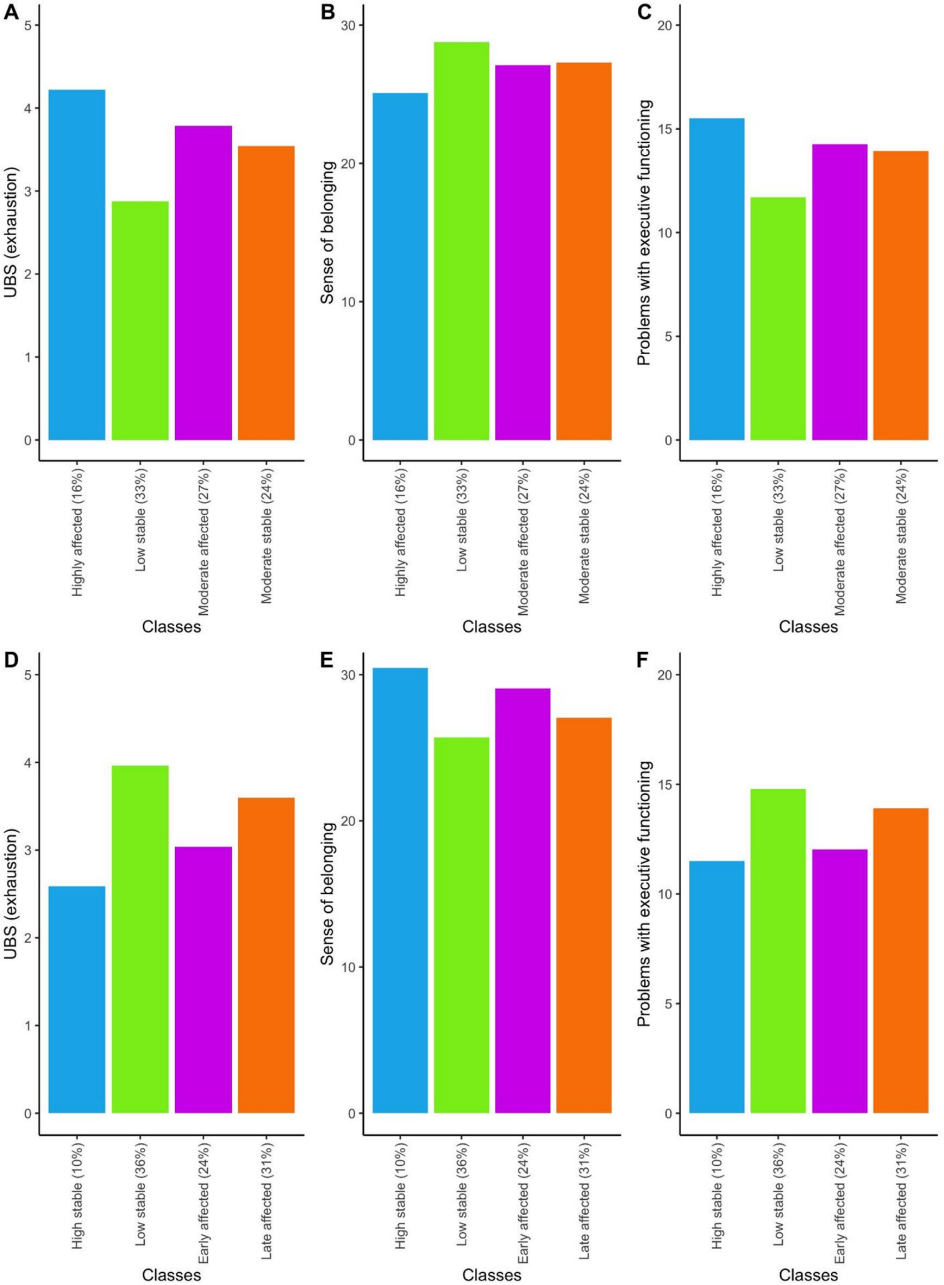
Protective and risk factors per class based on the negative mood (above) and vigor (below) trajectories.

The identified trajectories of mood based on negative mood and vigor were then compared on identifiable factors. The negative mood classes did not differ based on mean age at inclusion, gender, education, and percentage of academic drop-out (Table 2). However, significant differences were found for academic stress, sense of belonging and executive functioning. Academic stress was lower in the low stable negative mood class compared to the highly affected class, and the other two classes scored in between (Fig. 4; *F*(3) = 18.95, *p*<.001). The low stable class had a higher sense of belonging than the highly affected class, and the other two groups did not differ from either of them (*F*(3) = 4.74, *p*=.009). Finally, the low stable class showed less problems with executive functioning relative to the highly affected class and the other classes scored at an in between level (*F*(3) = 17.61, *p*<.001). Together, these findings show protective effects of sense of belonging and executive functions and accelerating effects of academic stress.

**Table 2:** Overview of demographic and protective or risk factors per class based on different trajectories of negative mood and Vigor during and after COVID-19 pandemic.

Classes based on negative mood	Highly affected	Low stable	Moderate stable	Moderate affected	*p*
N (%)	58 (16%)	119 (33%)	99 (27%)	87 (24%)	
Female (%)	84%	73%	81%	80%	1.00 (*X*(3) = 3.49)
Mean age at first wave of inclusion	18.16	17.79	17.64	18.00	1.00 (*F*(3) = 0.39)
Education					0.90 (*X*(18) = 22.15)
*Primary school*	0 (0%)	2 (2%)	0 (0%)	0 (0%)	
*High school*	30 (54%)	67 (56%)	55 (56%)	39 (45%)	
*Higher education*	26 (46%)	39 (33%)	36 (36%)	43 (49%)	
Academic dropout (%)	14%	13%	20%	13%	1.00 (*X*(3) = 2.2)
Psychiatric diagnosis at inclusion	23%	9%	14%	13%	0.59 (*X*(3) = 5.89)

*Classes based on vigor*	High vigor	Low vigor and improve	Middle vigor and improve	Middle and decreasing	*p*
N (%)	35 (10%)	131 (36%)	86 (24%)	111 (31%)	
Female (%)	57%	84%	71%	85%	0.002 (*X*(3) = 18.91)
Mean age at first wave of inclusion	16.19	18.36	17.73	17.90	1.00 (*F(*3) = 0.40)
Education					0.28 (*X*(18) = 28.35)
*Primary school*	1 (3%)	0 (0%)	1 (1%)	0 (0%)	
*High school*	25 (71%)	68 (52%)	46 (53%)	52 (47%)	
*Higher education*	7 (20%)	52 (40%)	32 (37%)	52 (47%)	
Academic dropout (%)	12%	15%	19%	14%	1.00 (*X*(3) = 1.41)
Psychiatric diagnosis at inclusion	9%	19%	5%	14%	0.18 (*X*(3) = 8.54)

The classes based on vigor differed on sex, as the high stable vigor class consisted of fewer females than the other three classes (*X*(3) = 18.91, *p*<.001) (Table 2). Academic stress was lower in the high stable vigor class and early affected classes compared to the other two classes (Fig. 4; *F*(3) = 17.08, *p*<.001). These classes were additionally characterized by higher sense of belonging (*F*(3) = 10.05, *p*<.001), and less executive functioning problems (*F*(3) = 13.79, *p*<.001). Together, these findings show a two-class differentiation in protective effects of sense of belonging and executive functions and accelerating effects of academic stress, rather than a gradual effect.

### Overlap between trajectories

**Fig. 5:**
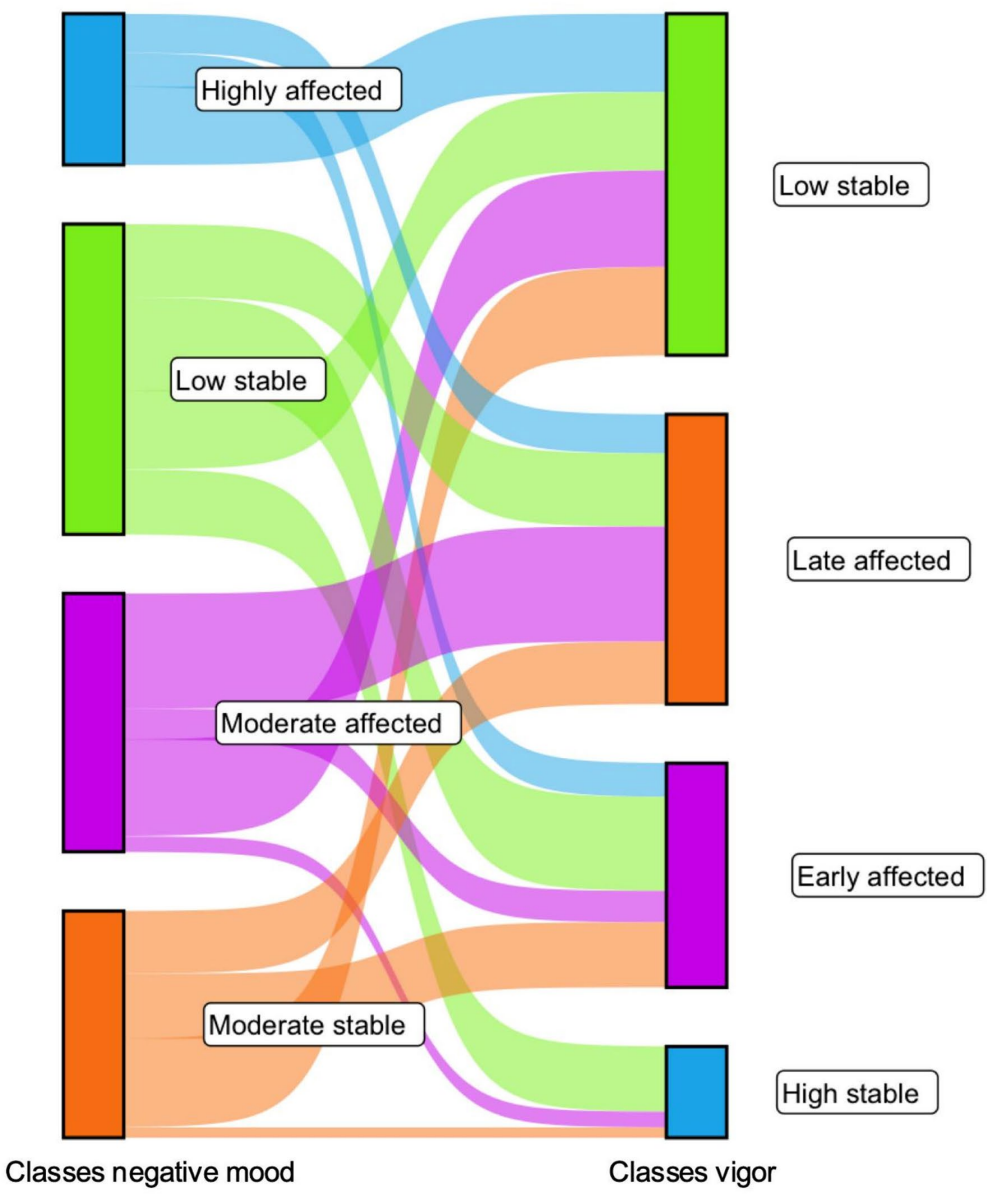
Overlap between classes based on negative mood development and vigor development.

To examine whether participants showed similar trajectories for negative mood and vigor, the overlap between the classes was studied. As can be seen in Fig. 5, there is variability in negative mood and vigor classes, confirming that these classes should be studied separately. The low stable negative mood class shows most overlap with the two highest vigor classes (high stable and early affected). The highly affected negative mood class showed most overlap with the low stable vigor class but not vice versa.

## Discussion

In the current study mood during and after the COVID-19 pandemic was examined in a longitudinal sample of adolescents aged 10–29. Prior research showed that this age group was relatively highly affected in terms of mental health problems compared to other age groups^45,46^. The aim of the study was to investigate in more detail whether there were distinct profiles in how adolescents’ mood responds to a stressful crisis. Consistent with prior research^6,9,47^, on a group level, adolescents showed an increase in negative mood during the COVID-19 pandemic, explained by both an increase in depression and tension, which declined after the pandemic restrictions had ended. Notably, adolescents’ feelings of tension remained relatively high after the restrictions during the pandemic had ended, whereas feelings of vigor gradually increased over time. This shows that adolescence is a vulnerable period, however, adolescents most likely differ in their patterns of resilience^11^. Indeed, we confirmed that the development of mood is better understood when adolescents were divided into subgroups, showing that different adolescents experienced different trajectories of mood over time. The current results should be interpreted with caution, as the observed changes in mood cannot be causally attributed to the pandemic.

Prior research in adolescents mostly studied average trajectories in mood during the COVID-19 pandemic. However, this does not consider how subgroups of adolescents deal with stressful situations, and what they might need in future crises. The subgroups of adolescents showed that the mood of a small majority of adolescents was not affected by the COVID-19 pandemic (57%). However, there were two groups who were significantly more affected by the COVID-19 pandemic and they only showed moderate levels of recovery. Contrary to earlier research, these subgroups only showed a decline in negative mood after the COVID-19 restrictions had finished, some even with a lingering effect^47^. Especially the adolescents who were highly affected (16%) that also showed a high average level of negative mood were characterized by a low sense of belonging, which might be a protector for the groups that showed resilience^33^. In addition, these adolescents showed more executive problems and higher academic stress. These higher levels of executive functioning problems could be a risk factor for negative mood^34^. It might be that less problems with executive functioning, could help adolescents with coping and therefore lead to higher levels of resilience^48^.

Adolescents also showed different trajectories regarding positive mood, specifically vigor, during and after the COVID-19 pandemic. The early affected group could be considered the most resilient, as they showed an initial effect of the COVID-19 pandemic on their vigor but recovered after restrictions had ended. This group, in addition to the high stable group, also showed the most promising protective measures, with high sense of belonging, low burnout and low levels of problems with executive functioning. This suggests that while it is common for adolescents to respond to stressful situations, those who show resilience effects, also experience protective effects of low academic stress, fewer executive functioning problems and more sense of belonging. It should be noted that sense of belonging was assessed post-pandemic, which can therefore not be interpreted as a predictive factor. Earlier research has shown that sense of belonging is relatively stable^49^, but in the current study it might have been affected by the pandemic.

The results are in line with earlier research in adults over the course of 1 year during the pandemic^29^. Here they also identified 4 groups, including a resilient group, an improving group, a worsening group, and a chronic group. The adults in the chronic group were characterized by more emotion regulation difficulties and a more challenging financial situation. However, they did not include data after the pandemic, which lacks the possibility to test how and whether adults recovered post-pandemic. We did not identify a group that showed chronic levels of negative mood that sustained after the pandemic. Although for the adolescents who were highly affected, their negative mood levels did not improve to be lower than those observed during the first COVID-19 lockdown. It should be noted that resilience is a multisystem construct; the current study examining mood trajectories focussed on individual psychological resilience, but future studies could also consider resilience in the adolescent’s social environment, or societal factors such as^50^.

It is important to consider that adolescents might respond differently than adults to the social restrictions, as adolescence is a time of social reorientation. During adolescence the influence of peers becomes increasingly important^51,52^. Adolescents spend more time with their peers and changes in social environment might contribute to the enhanced sensitivity of adolescents for social rejection by peers^53^. Thus, adolescence is a formative period for social processing, which might be dependent on the development of the social brain^54,55^. It is well known that even during pre-pandemic times, adolescents were at higher risk of developing mental health difficulties; 50% of all mental disorders are diagnosed before age 15 and almost 75% before age 25^56,57^. Therefore, it is important to monitor the development of mental health and wellbeing within this group, as they might be especially vulnerable for the effect of changes in the social environment on their mental wellbeing. Especially as they grew up in times of social restriction and lockdowns^5^. In addition, the current study showed that while some young people show individual resilience, others might need additional support from their social environments in challenging times.

The strengths of the current study included the large longitudinal data collection during and after the COVID-19 pandemic, which allowed us to study how and whether adolescents recovered, and the steps taken to test the reliability in more stringent and lenient datasets. However, some limitations should be considered. First, the study started after the COVID-19 pandemic had already begun, with the first wave of data collection taking place in May 2020 during the first lockdown (two months after the start of the pandemic). This means that pre-pandemic mood was not examined in this study. However, this limitation was mitigated by comparison to reports that showed an early pandemic effect on mood by comparing pre-and early pandemic levels^58^, by comparing psychiatric diagnosis at inclusion, and by having information on multiple waves of mood after the COVID-19 pandemic, which allowed us to study how adolescents’ mood recovered when the social situation returned to the pre-pandemic situation. Second, the current study examines mood trajectories, which is related to mental health^26^, but mood is subject to contextual factors (e.g., daily problems and sleep). Future studies could replicate these findings using questionnaires that assess mental health directly. Third, the classes found should be replicated in an independent sample, to examine their stability and replicability, also across different contexts (e.g., other regions). We conducted sensitivity analyses to examine the effects of sample, especially because adolescents with higher negative mood might show higher attrition rates. The lenient sample might therefore be more representative of the general population, in terms of gender, education and mood. The sensitivity analyses showed very similar results to the main analyses, with groups of adolescents who showed more stable mood, and groups that were affected but recovered after the pandemic had ended. Future sensitivity analyses could also focus on more specific age groups, such as early, middle or late adolescence, since the age range included in the current study is relatively large.

Interestingly, the groups identified based on negative mood were different from the groups based on positive mood. While there was some overlap, such as the low negative mood class overlapping with the classes with the higher vigor levels, the largest part of the groups did not overlap. This suggests that positive and negative mood are two separate processes, and not opposites on a spectrum, which is in line with previous studies suggesting them to be independent and having separable contributions to social functioning^59^. This is in line with the distinction between hedonic and eudaimonic well-being, as the reduction of negative affect (hedonic) does not automatically translate into heightened eudaimonic flourishing^60^. Therefore, future studies should also consider the resilience effect of vigor in stressful situations, as resilience in one domain does not imply resilience in another domain.

To conclude, this study showed that different adolescents had different trajectories of mood during and after the COVID-19 pandemic, arguing against a one-size fits all approach when providing support. Not every adolescent experiences and responds to a stressful situation similarly. Therefore, the effect of risk factors such as social restrictions on mental wellbeing should be monitored for potential future preventive strategies as it might differentially affect adolescents.

## Supplementary Information

Below is the link to the electronic supplementary material.

Supplementary Material 1

## Data Availability

The datasets used in the current study are available from the corresponding author on reasonable request. Code can be found on Zenodo (10.5281/zenodo.18494671).

## References

[1] Panchal, U. et al. The impact of COVID-19 lockdown on child and adolescent mental health: systematic review. *European Child and Adolescent Psychiatry*. 32 1151–1177 Preprint at (2023). 10.1007/s00787-021-01856-w 10.1007/s00787-021-01856-w PMC8371430 34406494

[2] Li, J. et al. Anxiety and depression among general population in China at the peak of the COVID-19 epidemic. *World Psychiatry*. 19 249–250 Preprint at (2020). 10.1002/wps.20758 10.1002/wps.20758 PMC7214959 32394560

[3] Bäuerle, A. et al. Increased generalized anxiety, depression and distress during the COVID-19 pandemic: A cross-sectional study in Germany. *J. Public. Health (United Kingdom)*. **42**, 672–678 (2020). 10.1093/pubmed/fdaa106 PMC7454766 32657323

[4] Brooks, S. K. et al. The psychological impact of quarantine and how to reduce it: rapid review of the evidence. *The Lancet*. 395 912–920 Preprint at (2020). 10.1016/S0140-6736(20)30460-8 10.1016/S0140-6736(20)30460-8 PMC7158942 32112714

[5] Orben, A., Tomova, L. & Blakemore, S. J. The effects of social deprivation on adolescent development and mental health. *The Lancet Child and Adolescent Health*. 4 634–640 Preprint at (2020). 10.1016/S2352-4642(20)30186-3 10.1016/S2352-4642(20)30186-3 PMC7292584 32540024

[6] Fischer, K. et al. Internalizing problems before and during the COVID-19 pandemic in independent samples of Dutch children and adolescents with and without pre-existing mental health problems. *Eur. Child. Adolesc. Psychiatry*. **32**, 1873–1883 (2023). 35616715 10.1007/s00787-022-01991-y PMC9133820

[7] Blakemore, S. J. Development of the social brain in adolescence. *Journal of the Royal Society of Medicine*. 105 111–116 Preprint at (2012). 10.1258/jrsm.2011.110221 10.1258/jrsm.2011.110221 PMC3308644 22434810

[8] Caspi, A. et al. Longitudinal assessment of mental health disorders and comorbidities across 4 decades among participants in the Dunedin birth cohort study. *JAMA Netw. Open.* **3**, e203221–e203221 (2020). 32315069 10.1001/jamanetworkopen.2020.3221 PMC7175086

[9] Barendse, M. E. A. et al. Longitudinal change in adolescent depression and anxiety symptoms from before to during the COVID-19 pandemic. *J. Res. Adolescence*. **33**, 74–91 (2023). 10.1111/jora.12781 PMC9349954 35799311

[10] De France, K., Hancock, G. R., Stack, D. M., Serbin, L. A. & Hollenstein, T. The mental health implications of COVID-19 for adolescents: Follow-Up of a Four-Wave longitudinal study during the pandemic. *Am. Psychol.* **77**, 85–99 (2021). 34110880 10.1037/amp0000838

[11] Masten, A. S. Resilience in children threatened by extreme adversity: frameworks for research, practice, and translational synergy. *Dev. Psychopathol.* **23**, 493–506 (2011). 23786691 10.1017/S0954579411000198

[12] Earvolino-Ramirez, M. Resilience: a concept analysis. *Nursing forum*. 42 73–82 Preprint at (2007). 10.1111/j.1744-6198.2007.00070.x 10.1111/j.1744-6198.2007.00070.x 17474940

[13] Toenders, Y. et al. Mood swings during development and their relation to sleep and brain development. *Sci Rep* 8537 (2024). 10.1038/s41598-024-59227-9 PMC11014928 38609481

[14] Penner, F., Hernandez Ortiz, J. & Sharp, C. *Change in youth mental health during the COVID-19 pandemic in a majority Hispanic/Latinx US sample*. *J Am. Acad. Child. Adolesc. Psychiatry*. **60**(4), 513–523 (2021). 10.1016/j.jaac.2020.12.027 33359408

[15] Pigaiani, Y. et al. Adolescent lifestyle behaviors, coping strategies and subjective wellbeing during the covid-19 pandemic: an online student survey. *Healthcare (Switzerland)* **8**, 472–484 (2020). 10.3390/healthcare8040472 PMC7712064 33182491

[16] Creswell, C. et al. Young people’s mental health during the COVID-19 pandemic. *The Lancet Child and Adolescent Health*. 5 535–537 Preprint at (2021). 10.1016/S2352-4642(21)00177-2 10.1016/S2352-4642(21)00177-2 PMC9765398 34174991

[17] Houghton, S. et al. Adolescents’ longitudinal trajectories of mental health and loneliness: the impact of COVID-19 school closures. *J. Adolesc.* **94**, 191–205 (2022). 35353417 10.1002/jad.12017 PMC9087620

[18] Pedrini, L. et al. Adolescents’ mental health and maladaptive behaviors before the Covid-19 pandemic and 1-year after: analysis of trajectories over time and associated factors. *Child Adolesc. Psychiatry Ment Health* **16**, 42 (2022). 10.1186/s13034-022-00474-x PMC9186010 35689203

[19] Fernández, R. S. et al. Psychological distress and mental health trajectories during the COVID-19 pandemic in argentina: a longitudinal study. *Sci Rep* **12**, 5632 (2022). 10.1038/s41598-022-09663-2 PMC8979149 35379888

[20] Wang, Y., Kala, M. P. & Jafar, T. H. Factors associated with psychological distress during the coronavirus disease 2019 (COVID- 19) pandemic on the predominantly general population: A systematic review and metaanalysis. *PLoS ONE*. 15 Preprint at (2020). 10.1371/journal.pone.0244630 10.1371/journal.pone.0244630 PMC7769562 33370404

[21] Manchia, M. et al. The impact of the prolonged COVID-19 pandemic on stress resilience and mental health: A critical review across waves. *European Neuropsychopharmacology*. 55 22–83 Preprint at (2022). 10.1016/j.euroneuro.2021.10.864 10.1016/j.euroneuro.2021.10.864 PMC8554139 34818601

[22] Fancourt, D., Steptoe, A. & Bu, F. Trajectories of anxiety and depressive symptoms during enforced isolation due to COVID-19 in england: a longitudinal observational study. *Lancet Psychiatry*. **8**, 141–149 (2021). 33308420 10.1016/S2215-0366(20)30482-X PMC7820109

[23] Janousch, C., Anyan, F., Morote, R. & Hjemdal, O. Resilience patterns of Swiss adolescents before and during the COVID-19 pandemic: a latent transition analysis. *Int. J. Adolesc. Youth*. **27**, 294–314 (2022).

[24] Musso, P. et al. The role of individual and contextual resources among Italian emerging adults during the COVID-19 pandemic: A Person- and Variable-Centered approach within the positive youth development framework. *Emerg. Adulthood*. **13**, 158–174 (2025).

[25] Shen, J. et al. Patterns and predictors of adolescent life change during the COVID-19 pandemic: a person-centered approach. *Curr. Psychol.* 2514–2528. 10.1007/s12144-021-02204-6/Published (2021). 10.1007/s12144-021-02204-6 PMC8435363 34539155

[26] Silk, J. S., Steinberg, L. & Morris, A. S. Adolescents’ emotion regulation in daily life: links to depressive symptoms and problem behavior. *Child. Dev.* **74**, 1869–1880 (2003). 14669901 10.1046/j.1467-8624.2003.00643.x

[27] Van de Groep, S., Zanolie, K., Green, K. H., Sweijen, S. W. & Crone, E. A. A daily diary study on adolescents’ mood, empathy, and prosocial behavior during the COVID-19 pandemic. *PLoS One* **15**, e0240349 (2020). 10.1371/journal.pone.0240349 PMC7540854 33027308

[28] Sawyer, S. M., Azzopardi, P. S., Wickremarathne, D. & Patton, G. C. The age of adolescence. *The Lancet Child and Adolescent Health*. 2 223–228 Preprint at (2018). 10.1016/S2352-4642(18)30022-1 10.1016/S2352-4642(18)30022-1 30169257

[29] Gambin, M. et al. Pandemic trajectories of depressive and anxiety symptoms and their predictors: Five-wave study during the COVID-19 pandemic in Poland. *Psychological Medicine*. 53 4291–4293 Preprint at (2023). 10.1017/S0033291721005420 10.1017/S0033291721005420 PMC8755528 34924069

[30] Guzman Holst, C. et al. Examining children and adolescent mental health trajectories during the COVID-19 pandemic: findings from a year of the Co‐SPACE study. *JCPP Advances* **3**, e12153 (2023). 10.1002/jcv2.12153 PMC10519733 37753152

[31] Green, K. H. et al. Mood and emotional reactivity of adolescents during the COVID-19 pandemic: short-term and long-term effects and the impact of social and socioeconomic stressors. *Sci Rep* **11**, 11563 (2021). 10.1038/s41598-021-90851-x PMC8172919 34078968

[32] Wu, L. et al. The associations of executive functions with resilience in early adulthood: A prospective longitudinal study. *J. Affect. Disord*. **282**, 1048–1054 (2021). 33601677 10.1016/j.jad.2021.01.031

[33] Scarf, D. et al. Somewhere I belong: Long-term increases in adolescents’ resilience are predicted by perceived belonging to the in-group. *Br. J. Soc. Psychol.* **55**, 588–599 (2016). 27448617 10.1111/bjso.12151

[34] Martel, M. M. et al. Childhood and adolescent resiliency, regulation, and executive functioning in relation to adolescent problems and competence in a high-risk sample. *Dev. Psychopathol.* **19**, 541–563 (2007). 17459183 10.1017/S0954579407070265

[35] Panagouli, E. et al. School performance among children and adolescents during covid-19 pandemic: A systematic review. *Children*. 8 Preprint at (2021). 10.3390/children8121134 10.3390/children8121134 PMC8700572 34943330

[36] Curran, S. L., Andrykowski, M. A. & Studts, J. L. Short form of the profile of mood States (POMS-SF): psychometric information. *Psychol. Assess.* **7**, 80–83 (1995).

[37] Green, K. H. et al. Socioeconomic hardship, uncertainty about the future, and adolescent mental wellbeing over a year during the COVID-19 pandemic. *Soc. Dev.* **32**, 1092–1114 (2023).

[38] Buchanan, T. et al. A short self-report measure of problems with executive function suitable for administration via the internet. *Behav. Res. Methods*. **42**, 709–714 (2010). 20805593 10.3758/BRM.42.3.709

[39] Schaufeli, W. & van Dierendonck, D. *Handleiding Van De Utrechtse Burnout Schaal (UBOS) (Manual Utrecht Burnout Scale)* (Swets Test Publishers Lisse, 2000).

[40] Carroll, A., Bower, J. M. & Muspratt, S. The conceptualization and construction of the self in a social Context—Social connectedness scale: A multidimensional scale for high school students. *Int. J. Educ. Res.* **81**, 97–107 (2017).

[41] Wood, S. N. *Generalized Additive Models: An Introduction with R, Second Edition*. *Generalized Additive Models: An Introduction with R, Second Edition* (2017). 10.1201/9781315370279

[42] Wardenaar, K. J. *Latent Class Growth Analysis and Growth Mixture Modeling Using R: A Tutorial for Two R-Packages and a Comparison with Mplus*. (2022).

[43] Van Buuren, S. & Groothuis-Oudshoorn, K. *Journal of Statistical Software Mice: Multivariate Imputation by Chained Equations in R*.. 45 (2011). http://www.jstatsoft.org/

[44] Proust-Lima, C., Philipps, V. & Liquet, B. Estimation of extended mixed models using latent classes and latent processes: the R package Lcmm. *J. Stat. Softw.* **78**, 1–56 (2017).

[45] Varma, P., Junge, M., Meaklim, H. & Jackson, M. L. Younger people are more vulnerable to stress, anxiety and depression during COVID-19 pandemic: A global cross-sectional survey. *Prog Neuropsychopharmacol. Biol. Psychiatry* **109**, 110236 (2021). 10.1016/j.pnpbp.2020.110236 PMC7834119 33373680

[46] Minihan, S. et al. Supplemental Material for Affect and Mental Health Across the Lifespan During a Year of the COVID-19 Pandemic: The Role of Emotion Regulation Strategies and Mental Flexibility. *Emotion* (2024). 10.1037/emo0001238.supp doi:10.1037/emo0001238.supp. 10.1037/emo0001238 PMC11064816 37199936

[47] Hawes, M. T., Szenczy, A. K., Olino, T. M., Nelson, B. D. & Klein, D. N. Trajectories of depression, anxiety and pandemic experiences; A longitudinal study of youth in new York during the Spring-Summer of 2020. *Psychiatry Res* **298**, 113778 (2021). 10.1016/j.psychres.2021.113778 PMC9754702 33550176

[48] Maciejewski, D., Brieant, A., Lee, J., King-Casas, B. & Kim-Spoon, J. Neural cognitive control moderates the relation between negative life events and depressive symptoms in adolescents. *J. Clin. Child. Adolesc. Psychol.* **49**, 118–133 (2020). 30084647 10.1080/15374416.2018.1491005 PMC6739181

[49] Ruedas-Gracia, N., Jiang, G. & Maghsoodi, A. H. Is belonging stable over time? A Four-Year longitudinal examination of university belonging differences among students. *Emerg. Adulthood*. **11**, 1022–1038 (2023).

[50] Liu, J. J. W., Reed, M. & Girard, T. A. Advancing resilience: an integrative, multi-system model of resilience. *Pers. Individ Dif*. **111**, 111–118 (2017).

[51] Brown, B., Bradford, S. & Petrie, S. The importance of peer group (‘crowd’) affiliation in adolescence. *J. Adolesc.* **9**, 73–96 (1986). 3700780 10.1016/s0140-1971(86)80029-x

[52] Nelson, E. E., Leibenluft, E., McClure, E. B. & Pine, D. S. The social re-orientation of adolescence: A neuroscience perspective on the process and its relation to psychopathology. *Psychological Medicine*. 35 163–174 Preprint at (2005). 10.1017/S0033291704003915 10.1017/s0033291704003915 15841674

[53] Sebastian, C., Viding, E., Williams, K. D. & Blakemore, S. J. Social brain development and the affective consequences of ostracism in adolescence. *Brain and Cognition*. 72 134–145 Preprint at (2010). 10.1016/j.bandc.2009.06.008 10.1016/j.bandc.2009.06.008 19628323

[54] Blakemore, S. J. & Mills, K. L. Is adolescence a sensitive period for sociocultural processing? *Annual Review of Psychology*. 65 187–207 Preprint at (2014). 10.1146/annurev-psych-010213-115202 10.1146/annurev-psych-010213-115202 24016274

[55] Crone, E. A. & Dahl, R. E. Understanding adolescence as a period of social-affective engagement and goal flexibility. *Nat. Rev. Neurosci.* **13**, 636–650 (2012). 22903221 10.1038/nrn3313

[56] Kim-Cohen, J. et al. Prior juvenile diagnoses in adults with mental disorder developmental Follow-Back of a Prospective-Longitudinal cohort. *Arch. Gen. Psychiatry*. **60**, 709–717 (2003). 12860775 10.1001/archpsyc.60.7.709

[57] Kessler, R. C. et al. Age of onset of mental disorders: A review of recent literature. *Curr. Opin. Psychiatry*. **20**, 359–364 (2007). 17551351 10.1097/YCO.0b013e32816ebc8c PMC1925038

[58] Barendse, M. E. A. et al. Neural correlates of self-evaluation in relation to age and pubertal development in early adolescent girls. *Dev Cogn. Neurosci* **44**, 100799 (2020). 10.1016/j.dcn.2020.100799 PMC7260676 32479376

[59] Bailen, N. H., Green, L. M. & Thompson, R. J. Understanding emotion in adolescents: A review of emotional Frequency, Intensity, Instability, and clarity. *Emot. Rev.* **11**, 63–73 (2019).

[60] Telzer, E. H., Fuligni, A. J., Lieberman, M. D. & Galvan, A. Neural sensitivity to eudaimonic and hedonic rewards differentially predict adolescent depressive symptoms over time. *Proceedings of the National Academy of Sciences* 111, 6600–6605 (2014). 10.1073/pnas.1323014111 PMC4020059 24753574

