# Supplementary material for: Mood trajectories showing resilience and recovery in young people during and after the COVID-19 pandemic
Source: Sci Rep. 2026 Feb 14;16:9108. doi: 10.1038/s41598-026-39808-6 (PMC12996328; doi:10.1038/s41598-026-39808-6)
Supplement: Supplementary file 1 — Supplementary Material 1 [file 41598_2026_39808_MOESM1_ESM.pdf]

Supplemental Material

Supplementary Figure S1. Flow chart of included participants in the current study. Participants were included based on age at inclusion (10-25 years), and availability of mood data on at least 4 waves.

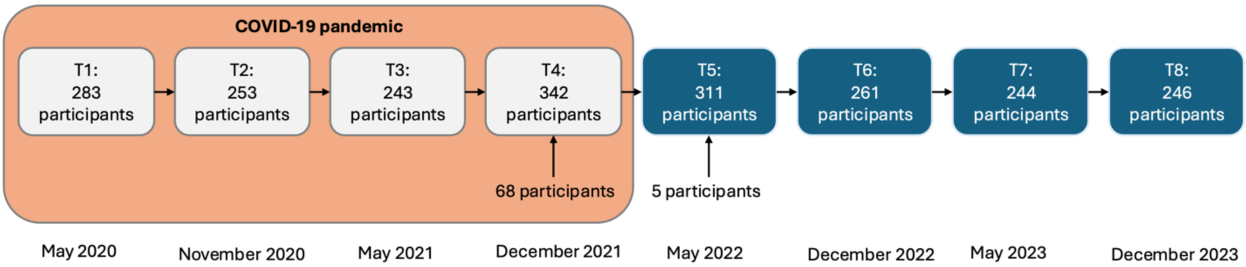

Supplementary Table S1. Results of the generalized additive mixed models (GAMMs)

Mood	k	edf	BIC	F	p-value
**Tension**	4	2.92	18263.43	16.99	<.001
**Depression**				19.86	<.001
	4	2.95	19628.34		
**Vigor**	3	1.00	16826.28	7.08	.02

Supplementary Figure S2. Correlation matrix

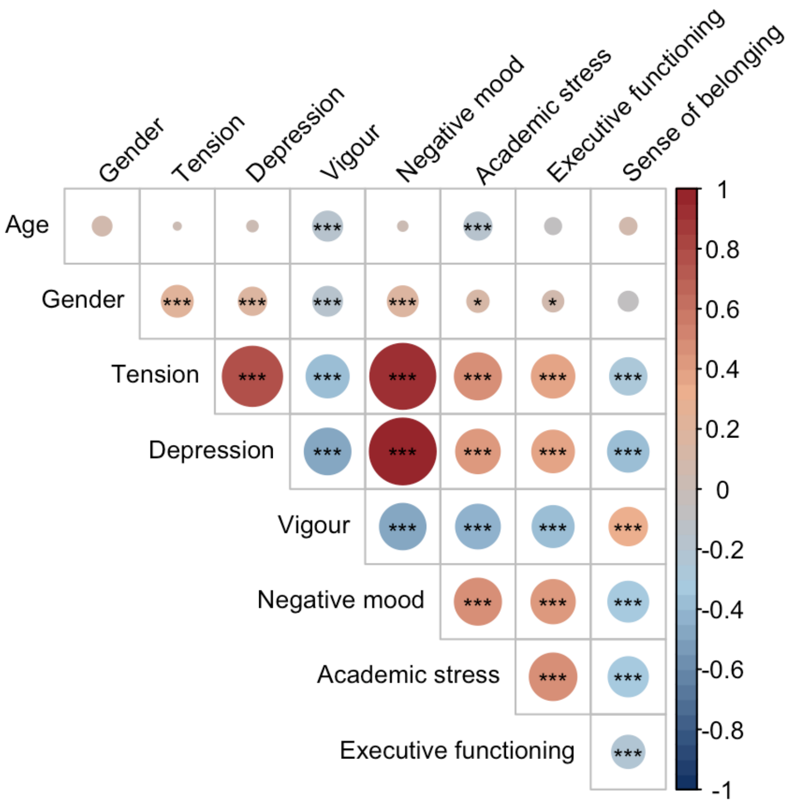

Supplementary Table S2. Fit measures of different number of classes in GMM for negative mood and vigor.

Number of classes	*AIC*	*BIC*	*Entropy*	*Class size (%)*	*Average posterior probability*	*LMRT p-value*
Negative mood						
1	9432.68	9607.92	1.00	100	1	
2	9399.88	9614.07	0.57	25.6,74.4	0.25,0.75	<.001
3	9372.09	9625.22	0.66	53.4,36.4,10.2	0.52,0.35,0.13	<.001
4	**9359.93**	**9652.02**	**0.70**	**32.8,16.0, 27.3,24.0**	**0.31,0.16, 0.28,0.25**	**<.001**
Vigor						
1	15295.86	15471.11	1	100	1	
2	15234.50	15448.69	0.63	33.9,66.1	0.34,0.66	<.001
3	15234.10	15487.23	0.59	32.0,41.3,26.7	0.31,0.38,0.30	.03
4	**15224.52**	**15516.60**	**0.69**	**9.6,36.1, 23.7,30.6**	**0.09,0.36, 0.23,0.32**	**<.001**

AIC: Akaike information criterion; BIC: Bayesian information criterion; LMRT: Lo–Mendell–Rubin test

Sensitivity analyses

To test the stability of the findings, the analyses were repeated with a more strictly and more lenient selected sample. For the strict dataset, only participants with data at 7 or more timepoints were included, which led to a sample of 147 participants (1176 observations). For the lenient dataset, participants with data on 2 or more timepoints were selected, leading to a sample of 915 participants (7320 observations). The demographic characteristics of the strict and lenient sample are displayed in Supplemental Table S2. The development of during and after the pandemic for all subscales is shown in Supplemental Figure S2. The pattern is similar as the pattern observed in the main analysis, though the lenient sample shows slightly less recovery after the pandemic in the negative mood subscales.

Supplemental Table S3. Overview of demographic of the strict and lenient sample across waves before imputation. Mean (SD) are being displayed unless otherwise specified.

	**Strict**	**Lenient**
**N**	147	915
**Demographics**		
Age at first inclusion	17.2 (3.2)	18.3 (3.2)
Gender (M/F/non-binary/other) *n* (%)	22 (15%) / 125 (85%)	259 (28%) / 636 (70%) / 10 (1%) / 10 (1%)
Ethnicity at first inclusion		

Dutch	96 (66%)	574 (63%)
Multiple ethnicities including Dutch	27 (18%)	177 (19%)
Education at first inclusion		
Primary school	1 (1%)	6 (1%)
High school VMBO	4 (3%)	13 (1%)
High school HAVO/VWO	91 (62%)	369 (40%)
Vocational education (mbo)	0 (0%)	188 (21%)
Higher education (hbo/university)	40 (27%)	142 (16%)
No current education / working	9 (6%)	42 (5%)
Academic drop-out (continued, repeated year, drop-out) *n* (%)	111 (76%) / 4 (3%) / 21 (14%)	610 (67%) / 22 (2%) / 47 (5%)
**Mood**		
Tension	6.8 (4.1)	7.5 (4.5)
Depression	6.1 (4.9)	7.2 (5.7)
Negative	12.9 (8.7)	14.7 (9.8)
Vigor	10.0 (3.3)	10.1 (3.4)
**Protective and risk factors**		
Utrecht Burnout Scale	3.5 (1.3)	3.4 (1.4)
Executive functioning	13.1 (3.8)	13.5 (4.0)
Sense of belonging	27.7 (5.1)	27.4 (5.0)

Note: Only gender, academic dropout, and education differed among samples (the main sample, lenient sample and strict sample).

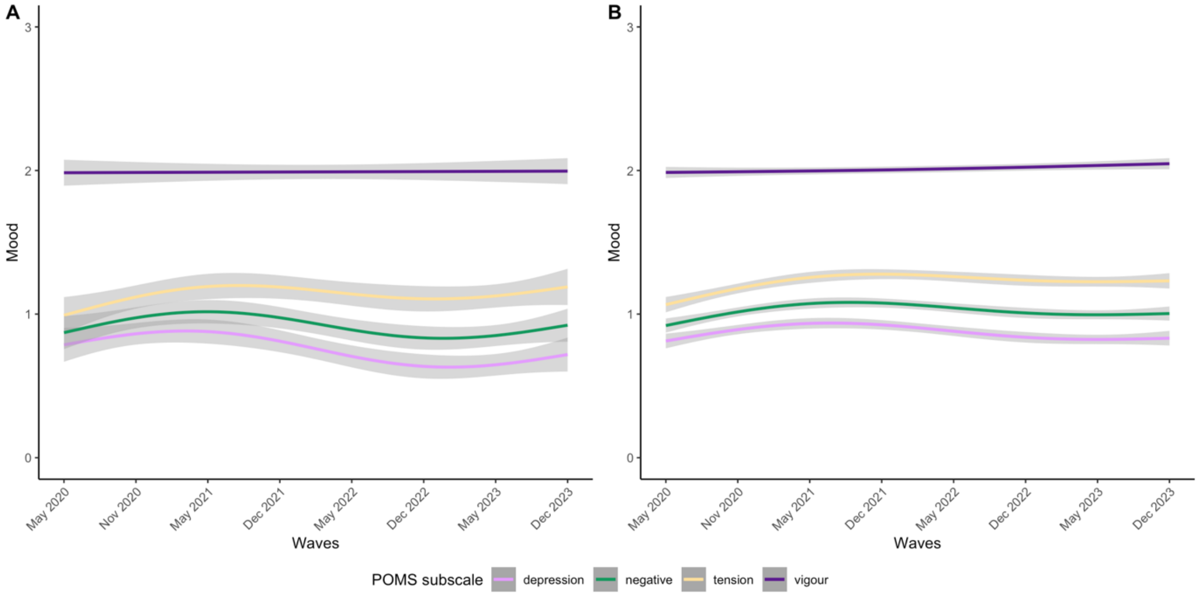

Supplemental Figure S3. Development of mood (per subscale) during and after the COVID-19 pandemic in the strict (A) and lenient (B) sample.

Sensitivity analyses – development of negative mood

Similar classes were identified in the strict and lenient sample as in the main analysis when examining the development of negative mood (Supplemental Figure S3). In all samples a highly affected and a low stable class were identified, though the highly affected class was smaller and more severe in the lenient sample. The lenient sample, like the main analysis also showed a moderate stable and moderate affected class. In the strict sample these classes were not identified, instead two early affected classes were found.

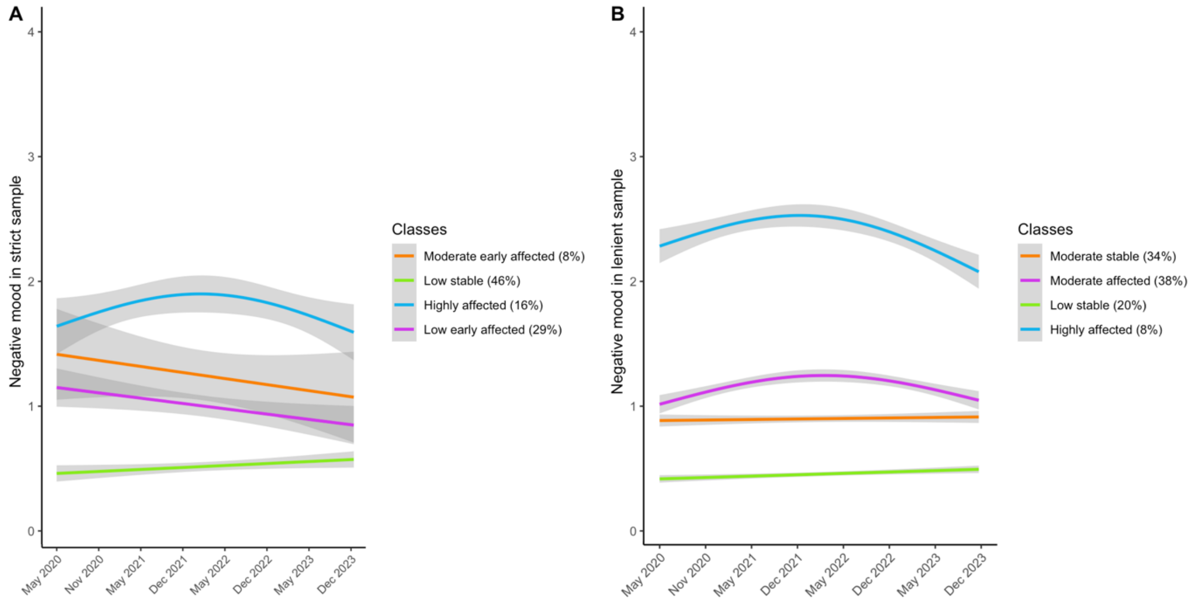

Supplemental Figure S4. Development of negative mood per class during and after the COVID-19 pandemic in the strict (A) and lenient (B) sample.

Sensitivity analyses – development of vigor

Similar to negative mood, the findings of the development of vigor were similar in the sensitivity analysis as in the main analysis (Supplemental Figure S4). In all samples a high stable and low stable vigor class were identified. In addition, in the both samples an early affected and late affected class were found, though they showed different levels of vigor at the end of the pandemic compared to in the main analysis.

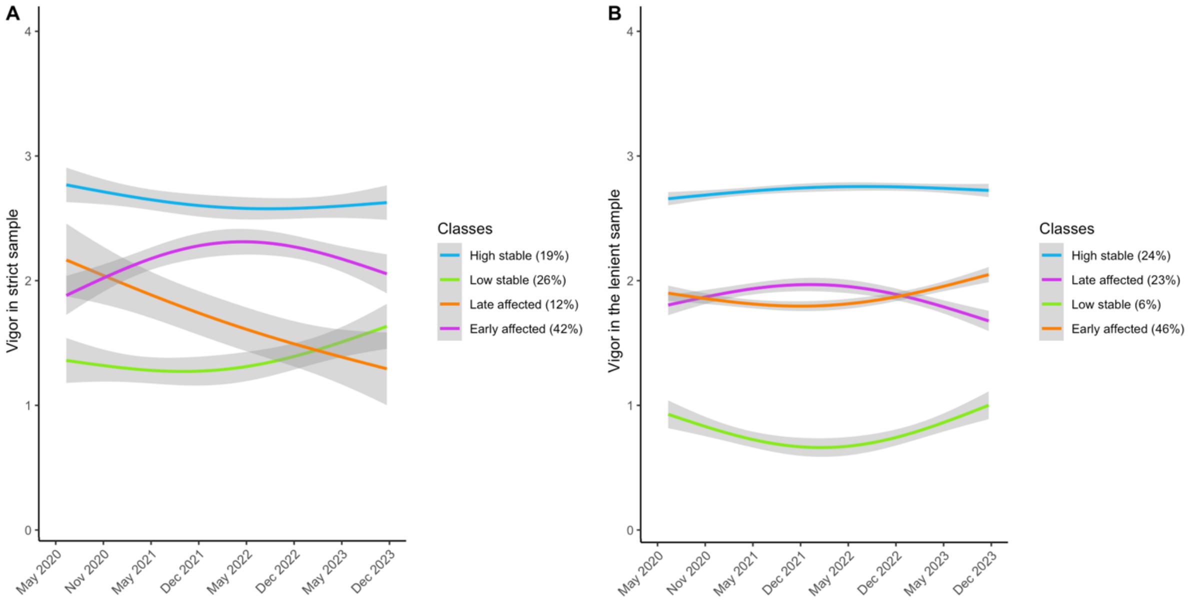

Supplemental Figure S5. Development of vigor per class during and after the COVID-19 pandemic in the strict (A) and lenient (B) sample.
